# Active elimination of intestinal cells drives oncogenic growth in organoids

**DOI:** 10.1016/j.celrep.2021.109307

**Published:** 2021-07-06

**Authors:** Ana Krotenberg Garcia, Arianna Fumagalli, Huy Quang Le, Rene Jackstadt, Tamsin Rosemary Margaret Lannagan, Owen James Sansom, Jacco van Rheenen, Saskia Jacoba Elisabeth Suijkerbuijk

**Affiliations:** 1 Department of Molecular Pathology, Oncode Institute, Netherlands Cancer Institute, Amsterdam 1066 CX, the Netherlands; 2 Cancer Research UK Beatson Institute, Glasgow G61 1BD, UK; 3 Institute of Cancer Sciences, University of Glasgow, Garscube Estate, Glasgow G61 1QH, UK; 4 Department of Immunology and Respiratory, Boehringer-Ingelheim Pharma GmbH & Co. KG, 88400 Biberach, Germany

**Keywords:** cell competition, small intestine, cancer, fetal-like, JNK, organoids

## Abstract

Competitive cell interactions play a crucial role in quality control during development and homeostasis. Here, we show that cancer cells use such interactions to actively eliminate wild-type intestine cells in enteroid monolayers and organoids. This apoptosis-dependent process boosts proliferation of intestinal cancer cells. The remaining wild-type population activates markers of primitive epithelia and transits to a fetal-like state. Prevention of this cell-state transition avoids elimination of wild-type cells and, importantly, limits the proliferation of cancer cells. Jun N-terminal kinase (JNK) signaling is activated in competing cells and is required for cell-state change and elimination of wild-type cells. Thus, cell competition drives growth of cancer cells by active out-competition of wild-type cells through forced cell death and cell-state change in a JNK-dependent manner.

## Highlights

• In organoids, wild-type intestine cells are actively eliminated by cancer cells
• Cell competition boosts proliferation of intestinal cancer cells
• Remaining wild-type cells adopt a fetal-like state
• JNK signaling in wild-type cells drives cell competition

## Graphical abstract

**Figure:**
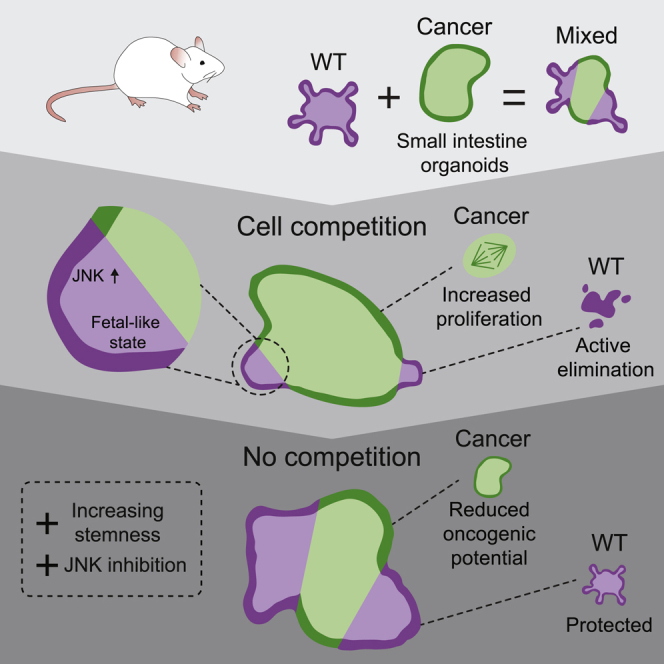

## Teaser

Interactions between cells helps coordinated decision making in tissues. Krotenberg Garcia et al. show that intestinal cancer cells use competitive cell interactions to actively eliminate surrounding wild-type cells. These interactions drive oncogenic growth in organoids.

## Introduction

Over the past years, it became evident that the internal proliferative potential of tumor cells is not sufficient for their expansion. Instead, tumor cells need to acquire multiple hallmarks of cancer, including growth-supporting interplay of tumor cells and their environment in order to sustain their proliferation (Hanahan and Weinberg, 2011). The basis of this interaction is often formed by processes that are, in origin, essential for normal early development and homeostasis (Suijkerbuijk and van Rheenen, 2017). One of those processes, cell competition, regulates survival of cells based on their relative fitness. In a homotypic context, cells strive and form viable tissues. However, in tissues built by heterogeneous populations, weaker cells will be removed by surrounding stronger cells. These features provide a strong mechanism that controls overall tissue and organismal fitness (Bowling et al., 2019; Clavería and Torres, 2016). Indeed, quality control by cell competition starts in the early mouse embryo (Clavería et al., 2013; Sancho et al., 2013) and continues to impact physiology up to late adulthood by determining the speed of aging (Merino et al., 2015).

In a tumor context, it has been shown that relative activation of YAP/TAZ, downstream effectors of the hippo signaling pathway, in peritumoral hepatocytes can influence growth of liver tumors by a process akin to cell competition (Moya et al., 2019). Furthermore, entosis, a form of cancer-driven cell competition, is correlated with a poor prognosis in patients with pancreatic ductal adenocarcinoma (Hayashi et al., 2020). These are examples of competitive cell interactions that suggest that cell competition could influence oncogenic growth. In addition, cell competition enforced by differential expression of isoforms of the protein Flower gives human cancer cells a competitive advantage over surrounding stromal tissue (Madan et al., 2019). We have shown that adenomas in the *Drosophila* midgut are dependent on active elimination of healthy surrounding tissue for their colonization (Suijkerbuijk et al., 2016). This illustrates that oncogenic growth can be driven by cell competition. However, the full potential and most of the mechanisms behind this process still need to be uncovered.

Here, we report that cell competition promotes growth of cancer cells in intestinal organoids. We show that cancer cells actively eradicate wild-type (WT) intestinal cells. Upon competition, JNK activation promotes a cell state transition in WT cells, which revert to a fetal-like state that is normally observed upon acute injury (Gregorieff et al., 2015; Nusse et al., 2018; Yui et al., 2018). Together, these competitional processes result in an increased colonization potential of cancer cells.

## Results

### Cancer cells out-compete WT small intestine cells

In order to investigate whether cell competition plays a role in mammalian intestinal cancer, we exploited the 3D organoid system (Sato et al., 2009), which closely resembles the architecture of the intestinal tissue. This allowed study of the interaction between cancer cells and WT cells in near-native conditions. Two different types of organoid cultures were derived from mouse small intestines: membrane-bound tdTomato-labeled WT cells and Dendra2-labeled intestinal cancer cells derived from Villin-Cre^ERT2^:*Apc*^fl/fl^*Kras*^G12D/WT^*Trp53*^fl/R172H^ transgenic mice (Fumagalli et al., 2017).

**Figure 1:**
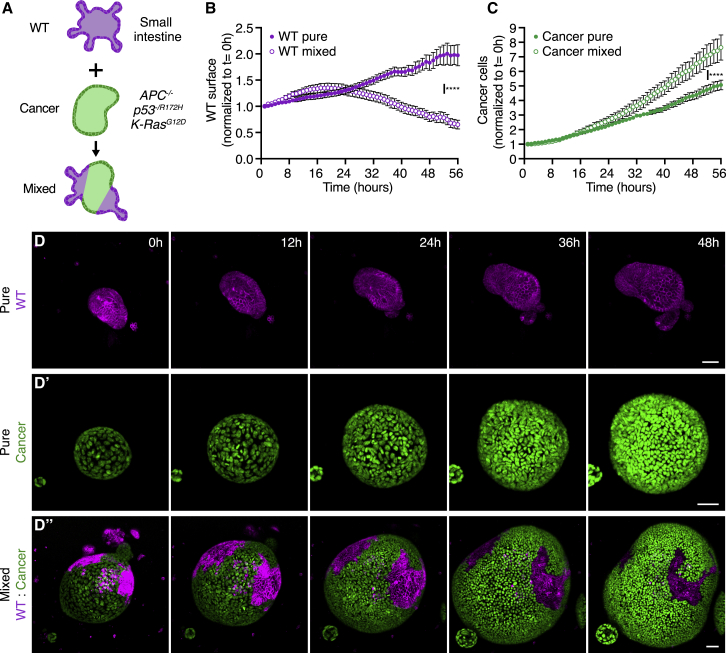
Cancer cells out-compete wild-type small intestine cells (A) Schematic depiction of a 3D model for cell competition in murine intestinal organoids. (B–D) Analysis of wild-type and cancer organoid growth under pure and mixed conditions by live imaging. Quantification of the wild-type cell surface (B) or number of cancer cells (C) within organoids normalized to the start of the time-lapse (mean ± SEM, paired t test, two-tailed; p < 0.0001, n = 12 and 15 organoids, B; p < 0.0001, n = 12 and 12, C). (D) Representative images of time-lapse series of pure wild-type (D), pure cancer (D’), and mixed (D’’) intestinal organoids; cancer cell nuclei are visualized by expression of H2B-Cerulean3. Scale bars represent 50 μm. See also Figure S1.

WT and cancer cell cultures were dissociated into small clumps of cells and concentrated to enable formation of mixed organoids (Figure 1A). Using time-lapse imaging of these cultures, we made two observations: (1) whereas pure WT cultures could expand over time (Figures 1B and 1D; Video S1), WT cells in mixed structures gradually disappeared (Figures 1B and 1D″; Video S3). (2) Tracking of the number of cancer cells by Histone H2B-Cerulean3 showed increased expansion in mixed compared to pure organoids (Figures 1C, 1D′, and 1D″; Videos S2 and S3). In addition, we adapted the recently described enteroid monolayer culture system that recapitulates all key aspects of the intestinal epithelium (Thorne et al., 2018) to study cell competition. For this, dissociated single cells were plated separately or mixed together on matrix coated imaging plates and followed for up to 10 days (Figure S1A). Pure cultures gradually covered the surface until a stable enteroid monolayer was formed (Figures S1B–S1D). However, once a full monolayer developed in mixed culture conditions, the surface area taken up by WT cells was not maintained but instead gradually decreased over time (Figures S1B’ and S1C). Furthermore, time-lapse imaging showed that WT cells reduced both in size and number when mixed with cancer cells (Figures S1E and S1E’; Video S4). Together, these data suggest that cancer cells can out-compete WT cells in mixed enteroid monolayer and organoid cultures.

Video S1. Time-lapse series of 3D reconstructed pure wild-type intestinal organoid, related to Figure 1D

Video S2. Time-lapse series of 3D reconstructed pure cancer intestinal organoid, related to Figure 1D′

Video S3. Time-lapse series of 3D reconstructed mixed intestinal organoid, related to Figure 1D″

Video S4. Time-lapse series of a competing enteroid monolayer, related to Figure S1E Arrow heads indicate examples of wild-type cells that are shrinking and being eliminated.

### Elimination of WT cells is driven by apoptosis

**Figure 2:**
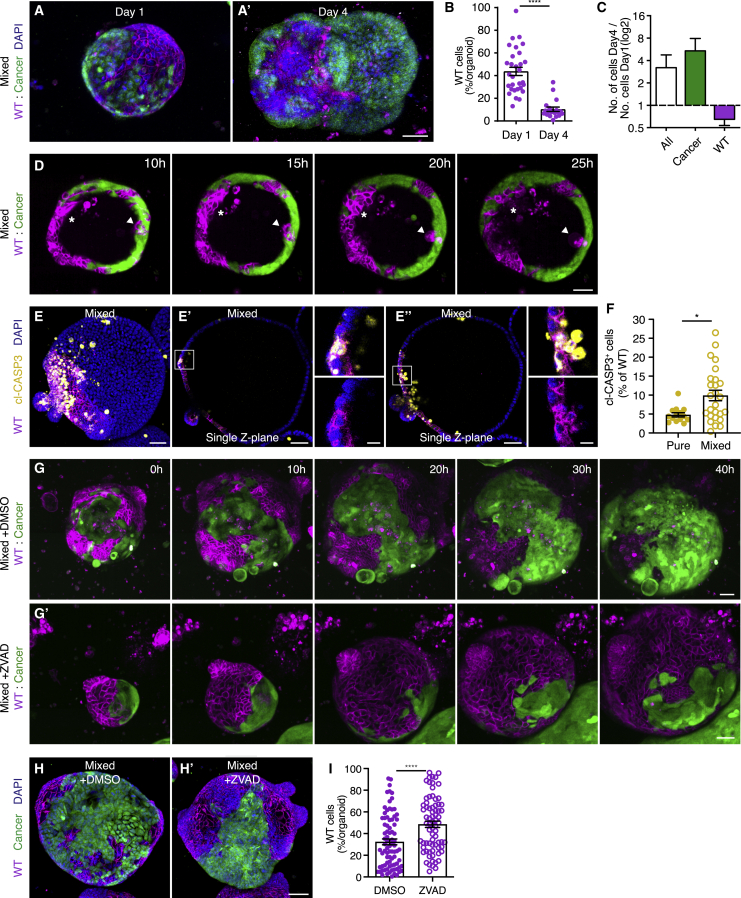
Elimination of wild-type cells is driven by apoptosis (A–C) Representative 3D-reconstructed confocal images of mixed organoids 1 day (A) and 4 days (A’) after plating; nuclei are stained with DAPI (blue). (B) The percentage of wild-type cells contributing to mixed organoids on day 1 and day 4 after plating is shown; each dot represents one organoid (mean ± SEM; Mann-Whitney; two-tailed; p < 0.0001; n = 30 and 19 organoids). (C) Displays the absolute number of cells in organoids shown in (B); the ratio of ‘day 4’ over ‘day 1’ of all (white), cancer (green), and wild-type (magenta) cells are plotted on a Log2 scale (mean ± SEM). (D) Representative images of time-lapse series of mixed intestinal organoid, maximum projection six Z stacks; extruding wild-type cells are indicated with an arrowhead (at interface with cancer cell surface) and asterisk (within the wild-type cell population). (E and F) Representative 3D-reconstructed (E) and single Z-plane (E’ and E’’) confocal images of a mixed organoid and quantification of the cl-CASP3^+^ cells relative to the total wild-type cell population (F). The organoids were stained for cl-CASP3 (yellow); nuclei are visualized with DAPI (blue). The insets display a 3.5× magnification of the area in the white box. Each dot in (F) represents one organoid (mean ± SEM; unpaired t test; two-tailed; p = 0.0120; n = 14 and 26 organoids). (G) Representative 3D-reconstructed confocal images of time-lapse series of control (G) and apoptosis-inhibited (G’) mixed intestinal organoids. (H and I) Representative 3D-reconstructed confocal image of control (H) and apoptosis-inhibited (H’) mixed organoids, nuclei are stained with DAPI (blue), and quantification of the percentage of wild-type cells contributing to mixed organoids (I); each dot represents one organoid (mean ± SEM; unpaired t test; two-tailed; p < 0.0001; n = 82 and 73 organoids). Scale bars represent 50 μm, excluding magnifications in (E), where scale bar represents 10 μm. See also Figures S1, S2, S4, and S5.

So far, we showed that the surface area of WT cells in mixed organoids declines over time, suggesting that they are eliminated by cancer cells. This was confirmed by a reduction in the percentage of WT cells in organoids from ±40% 1 day after mixing to ±10% on day 4 (Figures 2A and 2B). Additionally, we observed that the absolute number of WT cells that contributes to mixed organoids was lower 4 days after mixing although the number of total cells and of cancer cells increased (Figure 2C). This indicates that, although expansion of the WT cell population is slower than that of the cancer cell population, a difference in proliferation rate cannot be the sole determinant of the loss of WT cells.

In order to characterize how WT cells are lost from mixed organoids, we went back to time-lapse imaging. We observed two morphological changes that occurred to WT cells in mixed organoids compared to in pure organoids: the formation of typical crypt-villus structures was severely diminished and the extrusion of WT cells into the lumen of organoids increased (Figure 2D; Video S5). Interestingly, extrusion of WT cells was observed both at the interface with cancer cells (Figure 2D, arrowhead) and within the WT cell mass (Figure 2D, asterisk). Even though elimination of WT cells can be induced at a distance from cancer cells, multiple lines of evidence suggest that cell competition is short ranged: (1) WT and cancer cells are part of the same epithelium, and these competing cell populations directly interact (Figure S2A). (2) Occasional organoids of pure WT origin persist nearby mixed organoids (Figure S2B), suggesting that elimination of WT cells depends on intra-organoid interactions with cancer cells. (3) Growth and behavior of pure WT organoids plated in the same well with pure cancer or mixed organoids were undistinguishable of solely WT cultures (Figures S2C and S2D), indicating that the presence of both cell populations within the same organoid is required for out-competition of WT cells. It is important to note that this also shows that neither factors secreted by cancer cells nor depletion of components in the growth medium is sufficient to induce elimination of WT cells.

Video S5. Time-lapse series of mixed intestinal organoid, related to Figure 2D 3D reconstructed on the left, maximum projection of 6 Z stacks on the right. Cell extrusion is indicated with arrow heads.

We next asked whether programmed cell death is required for cancer-driven cell competition. We observed activation of caspase 3, a marker of apoptosis, in extruding WT cells (Figure 2E). This activation occurred both at the interface with cancer cells and further away and led to an overall increase in cell death in mixed WT cells (Figures 2E and 2F). Similarly, we observed increased rates of apoptotic WT cells in enteroid monolayers, although cancer cells are unaffected (Figures S1F–S1H). Surprisingly, treatment with the pan-caspase inhibitor Z-VAD-FMK could not prevent elimination of WT cells (Figures S1I and S1J), suggesting that a redundant process can cause out-competition of WT cells in these monolayers when apoptosis is inhibited. Potentially, this can be mediated by live cell extrusion, a mechanism involved in maintenance of cell density under homeostatic conditions (Eisenhoffer et al., 2012), but future work is required to determine involvement of this process. Importantly, time-lapse imaging of 3D cultures treated with Z-VAD-FMK showed increased maintenance of WT cells in mixed cultures (Figure 2G; Videos S6 and S7). Furthermore, quantification of the number of WT cells in mixed organoids showed that WT cells are not eliminated when apoptosis is inhibited (Figures 2H and 2I). It is interesting to note that the average percentage of WT cells in Z-VAD-FMK-treated mixed organoids is still lower (48.56%) than the expected percentage (66.67%) at the start of the experiment (based on the 2:1 starting ratio). This indicates that, although WT cells are not eliminated under these conditions, they do not gain the potential to out-compete cancer cells. Together, these data show that out-competition of WT cells is active and dependent on programmed cell death in 3D cultures.

Video S6. Time-lapse series of control treated 3D reconstructed mixed intestinal organoid, related to Figure 2G

Video S7. Time-lapse series of Z-VAD-FMK-treated 3D reconstructed mixed intestinal organoid, related to Figure 2G′

### Cancer cells boost their growth by cell competition

**Figure 3:**
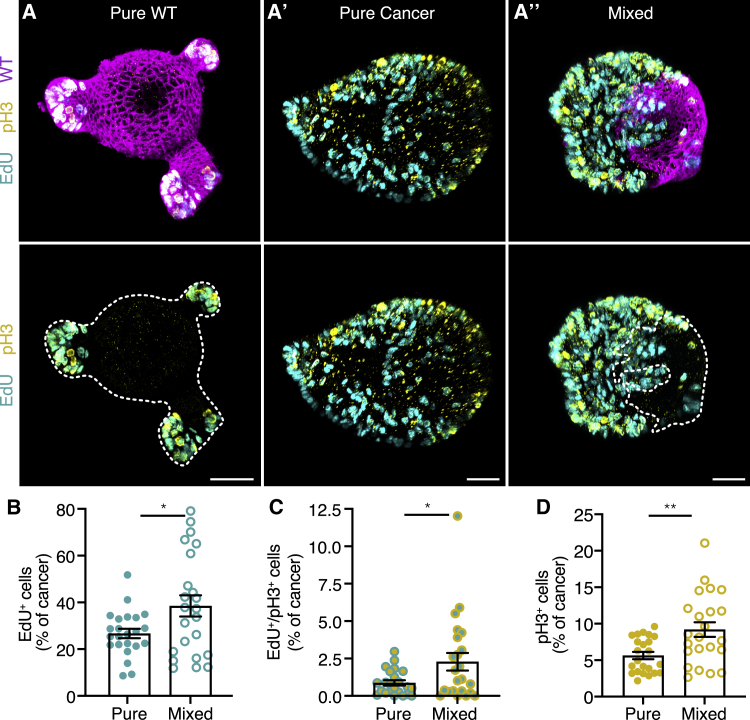
Cancer cells boost their growth by cell competition (A) Representative 3D-reconstructed confocal images of pure wild-type (A), pure cancer (A’), and mixed (A’’) organoids. Cells in S phase are labeled with EdU (cyan), and mitotic cells are marked by pH3 (yellow); cells that progressed from S phase to mitosis within 1 h are double positive. (B–D) Quantification of cancer cell proliferation in pure and mixed organoids. The number of cells in S phase (B), cells that progressed from S phase to mitosis within 1 h (C), and cells in mitosis (D) relative to the total cancer cell population is plotted. Each dot represents one organoid (mean ± SEM; unpaired t test; two-tailed; p = 0.0225, B; p = 0.0263, C; and p = 0.0027, D; n = 23 organoids for each condition). Scale bars represent 50 μm.

Previously, we observed increased expansion of competing cancer cells (Figure 1C) and therefore questioned whether cancer cells could be influenced by the presence of WT cells. First, we evaluated basal proliferation rates. By using markers that identify DNA replication (1-h EdU pulse) and active cell division (pH3), we could distinguish three populations of cells: cells in S phase (EdU^+^), cells that proceeded from S phase to mitosis (EdU^+^/pH3^+^), and mitotic cells (pH3^+^). Whereas proliferation in pure WT organoids was restricted to crypt regions (Figure 3A), no obvious spatial organization of proliferating cells was observed in pure cancer organoids (Figure 3A’). Importantly, competing cancer cells showed increased proliferation throughout the cell cycle (Figures 3A–3D). This implies that intestinal cancer cells boost their own proliferation and benefit from competitive cell interactions with WT intestinal cells.

### Cell competition induces a fetal-like state in WT cells

**Figure 4:**
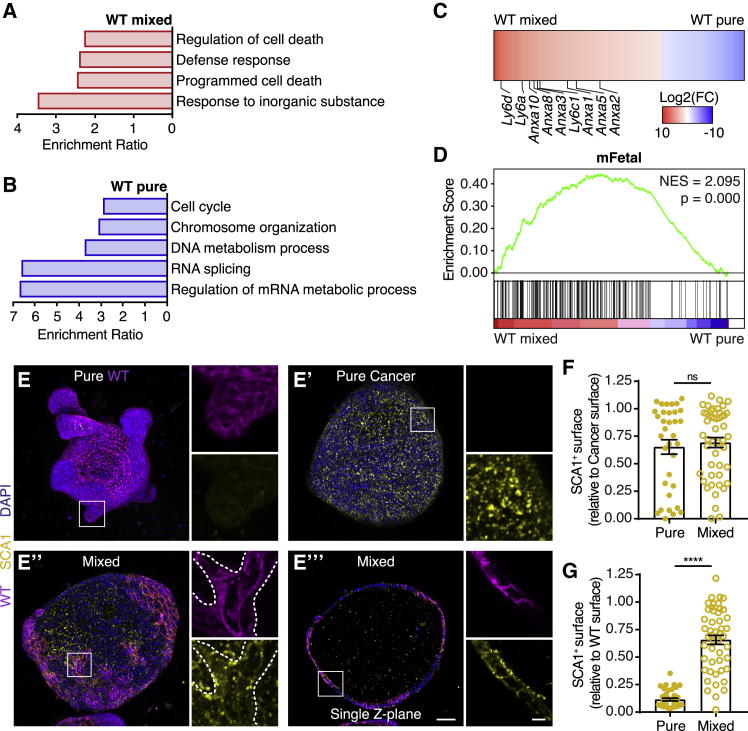
Cell competition induces a fetal-like state in WT cells (A and B) Gene Ontology analysis of differentially expressed genes (p < 0.05) in wild-type populations that are enriched in mixed (A) and pure (B) cells. (C) Heatmap of the fold change of genes that are differentially expressed in wild-type cells upon mixing (Log2). Genes of the *Ly6* and *Anxa* families are indicated. (D) Gene set enrichment analysis showing enrichment of a fetal signature (Yui et al., 2018) in mixed wild-type cells. (E–G) Representative 3D-reconstructed confocal images of pure WT (E), pure cancer (E’), mixed (E’’) organoids, and a single Z-plane of E’ (E’’’) and quantification of the SCA1^+^ surface relative to the total cancer (F) or wild-type (G) surface area. The organoids were stained for SCA1 (yellow); nuclei are visualized with DAPI (blue). The insets display a 3.5× magnification of the area in the white box. Each dot in (F) and (G) represents one organoid (mean ± SEM; non-parametric; ANOVA; multiple comparisons: p > 0.9999; n = 34 and 48 organoids, F; p < 0.0001, n = 39 and 48 organoids, G). Scale bars represent 50 μm, excluding magnifications in (E), where scale bar represents 10 μm. See also Figures S3 and S4.

In order to characterize molecular mechanisms underlying cell competition driven by cancer cells, we used bulk RNA sequencing to identify genes that are differentially expressed between pure and mixed organoids (Figures S3A–S3C). The transcriptome of competing cancer cells was very similar to that of pure cancer cells (Figure S3D), indicating that phenotypic changes in cancer cells induced by cell competition are not of a transcriptional nature. In contrast, the transcriptome of WT cells was dramatically changed upon cell competition (Figure S3D). Subsequent Gene Ontology analysis displayed enrichment of multiple cell-death-related pathways in competing WT cultures (Figure 4A), whereas processing of mRNA and cell proliferation were enriched in pure WT cultures (Figure 4B). These data confirm a negative impact of cell competition on WT cells. Interestingly, among the most highly upregulated genes were multiple members of the *Ly6* family (Figure 4C). The *Ly6* family genes are induced in intestinal epithelia after exposure to colitis (Flanagan et al., 2008), and one of its members, stem cell antigen-1 (*Sca1/Ly6a*), has recently been shown to be a marker of regenerating colonic epithelia (Yui et al., 2018). Furthermore, *Sca1* expression is activated in small intestinal epithelia that have been challenged with parasitic helminths (Nusse et al., 2018). Importantly, both injury responses cause a reprogramming of the tissue and adoption of an undifferentiated fetal-like state (Nusse et al., 2018; Yui et al., 2018). This response, which is essential for maintenance of the epithelial barrier in the intestine, is also characterized by increased expression of genes of the annexin family (Yui et al., 2018), which were also abundantly present among the highly upregulated genes in competing WT cells (Figure 4C). This prompted us to further investigate the exact transcriptional response induced in competing WT small intestinal cells, and we observed enrichment of the previously reported fetal-like and repair signatures (Figures 4D and S3E–S3G). This indicates that cancer cells actively damage the surrounding WT epithelium, which activates a response resembling an epithelium that is recovering from dextran sulfate sodium-induced colitis. Next, we validated the observed activation of the fetal-like response by immune-fluorescence staining of SCA1. A heterogeneous expression of SCA1 was detected in cancer cells, which was unaltered in mixed compared to pure cancer (Figures 4E and 4F), thereby reflecting the results of the transcriptional analysis. On the other hand, WT cells showed a homogeneous low expression of SCA1 in unchallenged conditions, which was dramatically increased in competing cells (Figures 4E and 4G). Thus, together, these data show that WT small intestine cells revert to a fetal-like state when challenged by competing cancer cells.

### Multiple types of intestinal cancer compete with WT cells

Over the past years, it became apparent that colorectal cancer can be stratified in multiple subtypes based on transcriptional profiling (Guinney et al., 2015). Each of these types display different molecular characteristics, which coincide with a different clinical progression of disease. So far, the here-described cell competition models were based on classical adenocarcinoma cells, resembling colorectal subtype 2. We next wondered whether other colorectal cancer subtypes can drive cell competition in intestinal organoids. Recently, epithelial NOTCH1 signaling has been shown to induce tumor microenvironment similar to human colorectal cancer subtype 4 (Jackstadt et al., 2019), and this is associated with an overall poor prognosis for patients. Organoids derived from these highly metastatic small intestinal tumors, induced by activation of KRas, together with deletion of p53 and overexpression of NOTCH1 intracellular domain (KPN), were mixed with WT small intestine cells. We found that KPN cancer cells can eliminate WT small intestine cells in an apoptosis-dependent manner (Figures S4A and S4B). In addition, cancer organoids derived from less invasive *Apc*^fl/+^*Trp53*^fl/fl^ Rosa26^N1icd/+^ (APN) small intestine tumors out-competed WT cells in mixed organoids (Figures S4C and S4D). Importantly, both types of intestinal cancer organoids could, like classical adenocarcinoma cells, induce activation of SCA1 in WT cells (Figures S4E–S4J). Thus, together, these data show that multiple types of intestinal cancer cells can induce a fetal-like state and eliminate WT cells.

### Loss of LGR5^+^ stem cells by induction of a fetal-like state through cell competition

**Figure 5:**
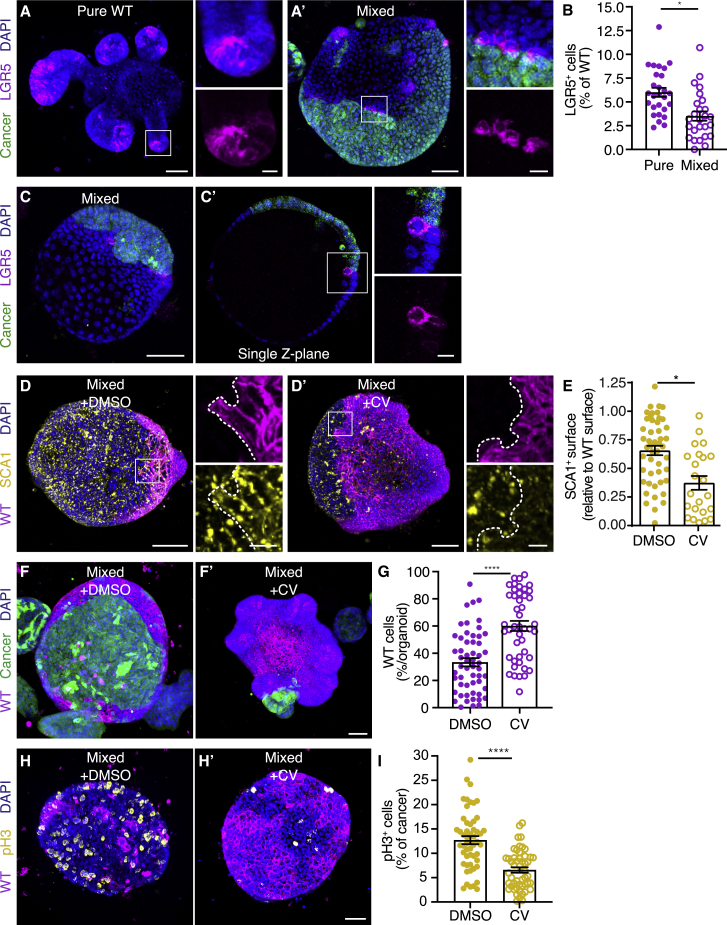
Increased stemness prevents cell competition (A–C) Representative 3D-reconstructed confocal images of pure WT (A) and mixed (A’ and C) organoids and single Z-plane of C (C’); LGR5^+^ intestinal stem cells (magenta) and nuclei (blue) are visualized. The insets display a 2× magnification of the area in the white box. (B) Graph displays a quantification of the number of LGR5^+^ cells relative to the total number of wild-type cells; each dot represents one organoid (mean ± SEM; one-way ANOVA; p = 0.0219; n = 27 and 26 organoids). (D and E) Representative 3D-reconstructed confocal images of control (D) and CV (D’) treated mixed organoids and quantification of the SCA1^+^ surface relative to the total wild-type surface area. The organoids were stained for SCA1 (yellow); nuclei are visualized with DAPI (blue). The insets display a 3.5× magnification of the area in the white box. Each dot in (E) represents one organoid (mean ± SEM; non-parametric; ANOVA; multiple comparisons: p = 0.0147; n = 48 and 23 organoids). Displayed control organoids are from the same dataset used in Figure 4G. (F and G) Representative 3D-reconstructed confocal image of control (F) and CV-treated (F’) mixed organoids, nuclei are stained with DAPI (blue), and quantification of the percentage of wild-type cells contributing to mixed organoids (G); each dot represents one organoid (mean ± SEM; unpaired t test; two-tailed; p < 0.0001; n = 55 and 45 organoids). (H and I) Representative 3D-reconstructed confocal images of control (H) and CV-treated (H’) mixed organoids. Mitotic cells are marked by pH3 (yellow) and nuclei with DAPI (blue). (I) Quantification of the number of pH3^+^ cells in mixed organoids relative to the total number of cancer cells; each dot represents one organoid (mean ± SEM; one-way ANOVA; p < 0.0001; n = 53 and 50 organoids). Scale bars represent 50 μm, excluding magnifications in (A), (C), and (D), where scale bar represents 10 μm. See also Figure S5.

The induction of a fetal-like state in adult intestinal epithelia has been reported to coincide with loss of intestinal stem cell (ISC) markers and removal of their niche (Nusse et al., 2018). We therefore next questioned how intestinal stem cells are affected by cancer-driven cell competition. ISCs are marked by leucine-rich-repeat-containing G-protein-coupled receptor 5 (LGR5) (Barker et al., 2007). We next derived organoids from Lgr5^DTR^ transgenic mice (Tian et al., 2011), in which the first coding exon of *Lgr5* was replaced with enhanced green fluorescent protein (EGFP) and human diphtheria toxin receptor (DTR). With the use of these Lgr5-DTR-EGFP organoids, we could detect ISCs localized in crypt regions of pure WT organoids (Figure 5A). Upon challenging these cells with competing cancer cells, we observed a marked decrease in the number of LGR5-positive cells (Figures 5A’ and 5B). We observed direct interaction of LGR5^+^ stem cells with cancer cells (Figure 5A’) and occasional extrusion of LGR5^+^ cells at this interface (Figure 5C). This suggests that the reduced population of LGR5^+^ cells is, at least in part, caused by elimination of these cells. However, because most of the WT population reverts to a fetal-like state (Figure 4G), the majority of eliminated WT cells are instead SCA1 positive (Figure S5A). Thus, cancer-driven cell competition induces a cell-state transition in the surrounding WT epithelium that is characterized by loss of LGR5^+^ stem cells and adoption of a fetal-like state.

### Increased stemness prevents cell competition

So far, we have shown that WT intestine cells undergo a cell-state transition when exposed to cancer cells. We next wondered whether reversal of this process could disrupt cell competition. Therefore, we sought a way to interfere with the cell state of WT organoids and turned to the previously described treatment with CHIR99021 and valproic acid (CV; Yin et al., 2014). This combined inhibition of glycogen synthase kinase 3β (GSK3β) and histone deacetylases (HDACs), reported to increase self-renewal of ISCs, indeed resulted in enrichment of LGR5-positive cells in cultures after 3 days of treatment (Figures S5B and S5C). This coincided with loss of expression of SCA1 in mixed WT cells (Figures 5D and 5E); thus, CV treatment can prevent the cell-state change induced by cell competition. Furthermore, increased stemness prevented loss of WT cells from mixed organoids (Figures 5F and 5G). Interestingly, the increased competitive potential of WT cells after CV treatment prevented the over-proliferation of cancer cells (Figures 5H and 5I). Because the mitotic index of pure cancer cultures was not decreased by CV treatment (Figure S5D), this was not a consequence of an autonomous effect of CV treatment on cancer cells. Thus, combined, these data suggest that increasing stemness of WT cells increases their competitive potential and prevents elimination.

### JNK signaling drives cell competition

**Figure 6:**
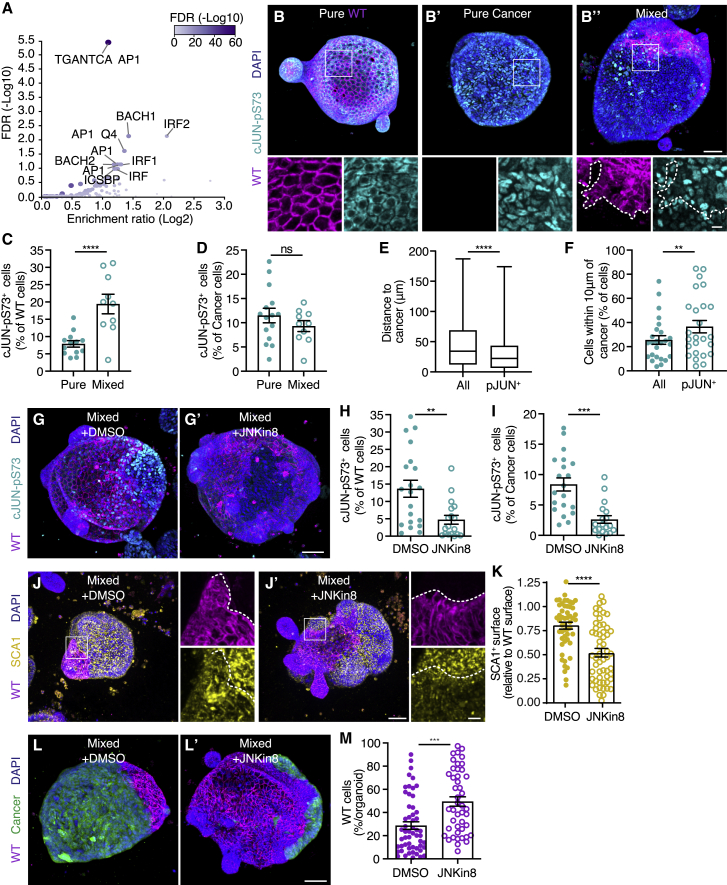
JNK signaling drives cell competition (A) Transcription factor target analysis of differentially expressed genes (p < 0.05) that are enriched in mixed wild-type cells. The graph displays enrichment (Log2) and false discovery rate (FDR) (−Log10) of gene sets (significantly enriched gene sets are indicated). (B) Representative 3D-reconstructed confocal images of pure WT (B), pure cancer (B’), and mixed (B’’) organoids, stained for activated cJUN (cyan); nuclei are visualized with DAPI (blue). The insets display a 3× magnification of the area in the white box. (C and D) Quantification of the number of cJUN-pS73^+^ cells relative to the total number of wild-type (C) and cancer (D) cells; each dot represents one organoid (mean ± SEM; one-way ANOVA; multiple comparisons; p < 0.0001, n = 13 and 10 organoids, C; p = 0.5740, n = 15 and 10 organoids, D). (E) Quantification of the distance of all (left) and cJUN-pS73^+^ (right) wild-type nuclei to the closest cancer cell surface in μm (median; 25^th^ to 75^th^ percentiles [box]; min. to max. [whiskers]; unpaired t test; two-tailed; p < 0.0001; n = 25 organoids). (F) Quantification of wild-type cells within a one-cell diameter (10 μm) of the closest cancer cell surface. The percentages of all (left) and cJUN-pS73^+^ (right) wild-type cells at the interface are displayed (mean ± SEM; paired t test; two-tailed; p = 0.0093; n = 25 organoids). (G–I) Representative 3D-reconstructed confocal images of control (G) and JNKin8 treated (G’) mixed organoids stained for activated cJUN (cyan); nuclei are visualized with DAPI (blue). (H and I) Quantification of the number of cJUN-pS73^+^ cells relative to the total number of wild-type (H) and cancer (I) cells; each dot represents one organoid (mean ± SEM; one-way ANOVA; multiple comparisons; p = 0.0041, n = 20 and 18 organoids, H; p = 0.0001, n = 20 and 18 organoids, I). (J and K) Representative 3D-reconstructed confocal images of control (J) and JNKin8 treated (J’) mixed organoids and quantification of the SCA1^+^ surface relative to the total wild-type surface area. The organoids were stained for SCA1 (yellow); nuclei are visualized with DAPI (blue). The insets display a 3.5× magnification of the area in the white box. Each dot in (K) represents one organoid (mean ± SEM; non-parametric; ANOVA; multiple comparisons: p < 0.0001; n = 52 and 56 organoids). (L and M) Representative 3D-reconstructed confocal image of control (L) and JNKin8 treated (L’) mixed organoids, nuclei are stained with DAPI (blue), and quantification of the percentage of wild-type cells contributing to mixed organoids (M); each dot represents one organoid (mean ± SEM; unpaired t test; two-tailed; p < 0.0001; n = 55 and 45 organoids). Scale bars represent 50 μm, excluding magnifications in (B) and (J), where scale bar represents 10 μm. See also Figure S6.

Next, we questioned which signaling pathways could control elimination and cell-state change of WT cells. Therefore, we performed a transcription factor target analysis on genes that were differentially expressed in bulk mRNA sequencing. This showed that Activator Protein 1 (AP-1) target sites were significantly enriched in genes that were higher expressed in competing WT cells (Figure 6A). AP-1 transcription factors are heterodimeric proteins that are activated upon exposure to numerous stressors, such as cytokines and hypoxia (Karin and Gallagher, 2005). To further characterize activation of AP-1, we analyzed phosphorylation of cJUN, an AP-1 family member (Minden et al., 1994). We observed increased nuclear signal of cJUN-pS73 in competing WT cells (Figures 6B and 6C), although activation was unchanged in cancer cells (Figures 6B and 6D). Interestingly, the distance of WT cJUN-pS73^+^ nuclei to cancer cells was smaller than the median distance of all WT nuclei (Figure 6E). Furthermore, there is an enrichment of WT cJUN-pS73^+^ nuclei at the interface with cancer cells (Figure 6F). Together, this suggests that short-ranged interactions with cancer cells promote activation of cJUN in WT cells. This phosphorylation site is a substrate of the JNK, which is activated in competing WT cells (Figure S6). JNK signaling plays a critical role in controlling both cell proliferation and death (Karin and Gallagher, 2005; Minden et al., 1994) and is a key regulator in many forms of cell competition (Tamori and Deng, 2011), including tumor-induced cell competition in the *Drosophila* adult intestine (Suijkerbuijk et al., 2016). We used treatment with the selective JNK inhibitor JNK-IN-8 (Zhang et al., 2012) to interfere with overall JNK signaling, which indeed prevented phosphorylation of cJUN in WT and cancer cells (Figures 6G–6I). Furthermore, treatment with JNK-IN-8 significantly reduced SCA1 levels in WT cells (Figures 6J and 6K), suggesting that active JNK signaling is required for the cell-state transition that is enforced by cell competition. Importantly, we found that inhibition of JNK prevents elimination of WT cells (Figures 6L and 6M). Thus, JNK is activated in competing cells and is required for eradication and cell-state transition of WT small intestine cells.

### JNK activity in WT cells is required for cell competition

**Figure 7:**
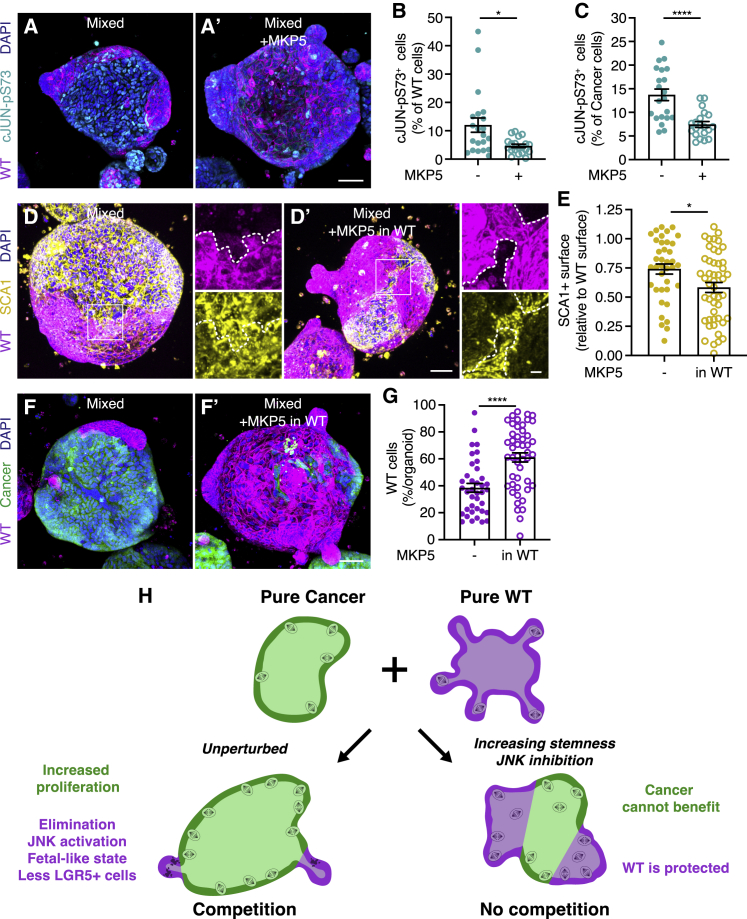
JNK activity in wild-type cells is required for cell competition (A–C) Representative 3D-reconstructed confocal images of control (A) and MKP5-expressing (A’) mixed organoids stained for activated cJUN (cyan); nuclei are visualized with DAPI (blue). (B and C) Quantification of the number of cJUN-pS73^+^ cells relative to the total number of wild-type (B) and cancer (C) cells; each dot represents one organoid (mean ± SEM; one-way ANOVA; multiple comparisons; p = 0.0156, n = 21 and 20 organoids, B; p < 0.0001, n = 21 and 20 organoids, C). (D and E) Representative 3D-reconstructed confocal images of control (D) and doxycycline-treated (D’) mixed organoids formed by doxycycline-inducible MKP5 wild-type and control cancer cells. The organoids were stained for SCA1 (yellow); nuclei are visualized with DAPI (blue). The insets display a 2.5× magnification of the area in the white box. (E) Quantification of the SCA1^+^ surface relative to the total wild-type surface area; each dot represents one organoid (mean ± SEM; non-parametric; ANOVA; multiple comparisons: p = 0.0129; n = 38 and 48 organoids). (F and G) Representative 3D-reconstructed confocal image of control (F) and doxycycline-treated (F’) mixed organoids formed by doxycycline-inducible MKP5 wild-type and control cancer cells, nuclei are stained with DAPI (blue), and quantification of the percentage of wild-type cells contributing to mixed organoids (G); each dot represents one organoid (mean ± SEM; unpaired t test; two-tailed; p < 0.0001; n = 38 and 48 organoids). (H) Schematic model depicting increased growth of intestinal tumor cells driven by cell competition in murine organoids. Cancer cells induce active elimination and cell fate transition of wild-type cells, while fueling their oncogenic growth. Scale bars represent 50 μm, excluding magnifications in (D), where scale bar represents 10 μm. See also Figure S7.

Next, in order to untangle where JNK activation is required, we sought a manner to specifically inhibit signaling in individual cell populations. Mitogen-activated protein kinase phosphatase 5 (MKP5) (also known as DUSP10) is a member of the dual-specificity phosphatase family that inactivates JNK and p38 *in vitro* (Theodosiou et al., 1999; Tanoue et al., 1999) and regulates JNK activity in mouse cells (Zhang et al., 2004). We observed that doxycycline-inducible expression of MKP5, detected by co-expression of mTurquoise2 (Figure S7A), results in reduced activation of cJUN in WT and cancer cells (Figures 7A–7C). This confirms that MKP5 inactivates JNK signaling in mouse intestinal organoids. Expression of MKP5 solely in cancer cells did not affect SCA1 activation (Figures S7B and S7C) or prevent elimination of WT cells (Figures S7D and S7E). However, specific expression of the phosphatase in only WT cells inhibited SCA1 expression (Figures 7D and 7E) and, importantly, rescued WT cells (Figures 7F and 7G). Together, this shows that induction of a cell-state transition and out-competition of WT cells is dependent on JNK activity in WT cells.

## Discussion

Effects of competition on cell fate have been shown in many tissues. Classical examples are neutral drift in the intestinal stem cells niche (Lopez-Garcia et al., 2010; Snippert et al., 2010) and maintenance of stem cells in the *Drosophila* testis (Sheng et al., 2009). A dual effect of cell competition on weaker cell populations, through active elimination by cell death combined with reduced stem cell renewal, has been observed under homeostasis. Both in the adult *Drosophila* midgut and in developing mouse skin, weaker cells, induced by ribosome impairment or reduced expression of *mycn*, are removed from the tissue by stronger cells through apoptosis and forced differentiation (Ellis et al., 2019; Kolahgar et al., 2015). Here, we provide an example of a combined mechanism of forced elimination and cell-state transition in relation to cancer (Figure 7H).

Many studies have reported that tumor growth is highly context dependent. In particular, a strong correlation exists between inflammation and intestinal cancer. For example, patients with Crohn’s disease have 20–30 times higher risk of developing adenocarcinomas in the small intestine and inflammatory bowel disease is a strong risk factor for colorectal cancer (Beaugerie and Itzkowitz, 2015). Similarly, in mouse models of intestinal cancer, formation of colonic polyps is strongly enhanced by inflammation induced by infection with enterotoxigenic *Bacteroides fragilis* or treatment with dextran sodium sulfate (Tanaka et al., 2006; Wu et al., 2009). Interestingly, these are conditions in which a fetal-like response is activated in the intestine (Gregorieff et al., 2015; Nusse et al., 2018; Yui et al., 2018), similar to the here-reported primitive state induced upon cancer-driven cell competition. Future efforts should be directed toward increasing understanding of this connection of a fetal-like state and tumorigenesis.

Under normal circumstances, the fetal-like response promotes regeneration of the intestinal tissue. A recent study describing the kinetics of regeneration after removal of a damaging insult has shown that reformation of a homeostatic intestinal epithelium takes approximately 3 weeks (Wang et al., 2019). Therefore, during chronic exposure of the epithelium to an insult, such as close proximity of a tumor, healthy tissue will never be allowed to fully recover. This response is therefore counterproductive under the circumstances described here. Interestingly, induction of a fetal-like state upon injury is not restricted to the intestinal epithelium and is also observed in multiple other tissues (Fernandez Vallone et al., 2016; Gadye et al., 2017; Lin et al., 2017). This suggests that our observation that tumors can push surrounding WT tissue in a primitive state could be more universal.

Cell competition can be tumor suppressive; for example, cells expressing oncogenic H-Ras are eliminated from intestinal and pancreatic epithelia through apical extrusion (Kon et al., 2017; Sasaki et al., 2018). Furthermore, WT cells actively eliminate mutant aberrant foci in the skin (Brown et al., 2017). However, this effect is not solely determined by autonomous properties of the tumor but is highly context dependent. For instance, obesity induced by a high-fat diet prevents cell-competition-driven elimination of oncogenic cells (Sasaki et al., 2018). This illustrates how the surrounding environment dictates behavior of tumors and that tumor fitness, and thus its oncogenic potential, can be changed by external stimuli. Here, we report that JNK signaling is a major regulator of WT cell elimination and thus overall fitness of competing healthy cells. This may open up new options of treatment, where promoting fitness of the host tissue, through JNK inhibition, can help to tip the balance toward tumor-suppressive cell competition.

## STAR★Methods

### Key resources table

**Table:** 

REAGENT or RESOURCE	SOURCE	IDENTIFIER
**Antibodies**		

anti-Cleaved Caspase-3 (Asp175)	Cell Signaling	9661; RRID:AB_2341188
anti-phospho-Histone H3 (Ser10)	Millipore	06-570; RRID:AB_310177
anti-GFP	Abcam	ab6673; RRID:AB_305643
anti-Phospho-c-Jun (Ser73) (D47G9)	Cell Signaling	3270; RRID:AB_2129575
anti-Phospho-JNK1+JNK2 (T183 + Y185)	Abcam	ab4821; RRID:AB_2141012
anti-Ly-6A/E (Sca-1)	Biolegend	108101; RRID:AB_313338
Chicken anti-Rat, Alexa Fluor 647	ThermoFisher Scientific	A21472; RRID:AB_2535875
Donkey anti-Goat, Alexa Fluor 555	ThermoFisher Scientific	A32816; RRID:AB_2762839
Chicken anti-Rabbit, Alexa Fluor 647	ThermoFisher Scientific	A21443; RRID:AB_2535861
Donkey anti-Rabbit, Alexa Fluor 568	ThermoFisher Scientific	A10042; RRID:AB_2534017
Donkey anti-Goat, Alexa Fluor 488	ThermoFisher Scientific	A11055; RRID:AB_2534102

**Chemicals, peptides, and recombinant proteins**		

Advanced DMEM F/12	ThermoFisher Scientific	12634-010
B27	ThermoFisher Scientific	17504-044
Cultrex PathClear Reduced Growth Factor Basement Membrane Extract Type 2	R&D Systems	3533-005-02
N-acetylcysteine	Sigma-Aldrich	A9165
TryplE	Thermo Fisher Scientific	12605-010
Dapi	Thermo Fisher Scientific	D1306
Phalloidin Alexa Fluor 647	Thermo Fisher Scientific	A22287
GlutaMAX	Thermo Fisher Scientific	35050-068
HEPES	Thermo Fisher Scientific	15630-056
Penicillin/streptomycin	Thermo Fisher Scientific	15140-122
mEGF	Peprotech	315-09
Noggin	prepared in house	n/a
R-spondin1	prepared in house	n/a
Y-27632	Abmole	M1817
CHIR-99021	Tocris	4423
Valproic acid	Sigma Aldrich	PHR1061-1G
JNK-IN-8	Sigma Aldrich	SML1246
Doxycycline	Sigma Aldrich	D9891

**Critical commercial assays**		

Click-iT EdU Cell Proliferation Kit for Imaging Alexa Fluor 647	ThermoFischer Scientific	C10340

**Deposited data**		

mFetal	(Yui et al., 2018)	N/A
mRepair	(Yui et al., 2018)	N/A
RNA-seq data	NCBI GEO	GEO: GSE176027

**Experimental models: cell lines**		

Villin-Cre^ERT2^*Apc*^fl/fl^*Kras*^G12D/WT^*Tr53*^fl/R172H^	(Fumagalli et al., 2017).	N/A
Villin-Cre^ERT2^*Kras*^G12D/WT^*Trp53*^fl/fl^Rosa26^N1icd/+^	ACRCelerate Colorectal Cancer Stratified Medicine Network Consortium (Jackstadt et al., 2019)	N/A
Villin-Cre^ERT2^*Apc*^fl/+^*Trp53*^fl/fl^Rosa26^N1icd/+^	ACRCelerate Colorectal Cancer Stratified Medicine Network Consortium (Jackstadt et al., 2019)	N/A

**Experimental models: organisms/strains**		

*Mus musculus*_Lgr5DTR-eGFP	Genentech (Tian et al., 2011)	N/A
*Mus musculus*_*Gt(ROSA)* *26Sor*^*tm4(ACTB-tdTomato,-EGFP)Luo*^/J	The Jackson Laboratory	007676
*Mus musculus*_eCadherin-mCFP	(Snippert et al., 2010)	N/A

**Recombinant DNA**		

A dual lentiviral vector 3rd generation Tet-regulatory protein expression system for doxycycline-inducible expression of MKP5	VectorBuilder Inc	N/A
pLentiPGK Hygro DEST H2B-mCerulean3	Addgene	90234
pMDLg/pRRE	Addgene	12251
pRSV-Rev	Addgene	12253
pMD2.G	Addgene	12259

**Software and algorithms**		

Imaris	Oxford Instruments	9.3.1
FIJI	https://imagej.net	2.1.0/1.53h
Prism	GraphPad	9.0.0 (86)
ZEN	Zeiss	2.6 (Blue edition)
FlowJo 10.6.1	BD Biosciences	https://www.flowjo.com/
R software	GNU project	https://www.r-project.org/

**Other**		

ibiTreat #1.5 polymer coverslip 96 Well μ-Plate	IBIDI	89626
μ-Slide 8 Well chambered slides	IBIDI	80827
glass-bottom 96 well SensoPlate	Greiner Bio-One	655892

### Resource availability

#### Lead contact

Further information and requests for resources and reagents should be directed to and will be fulfilled by the Lead Contact, Saskia J.E. Suijkerbuijk (s.suijkerbuijk@nki.nl).

#### Materials availability

All unique/stable reagents generated in this study are available from the Lead Contact with a completed Materials Transfer Agreement.

#### Data and code availability

All used software is listed in the Key resources table. The data discussed in this publication have been deposited in NCBI’s Gene Expression Omnibus (Edgar, Domrachev and Lash, 2002) and are accessible through GEO Series accession number GEO: GSE176027 (https://www.ncbi.nlm.nih.gov/geo/query/acc.cgi?acc=GSE176027)

### Experimental model and subject details

#### Isolation wild-type small intestine organoids

All experiments were performed in accordance with the Animal Welfare Committee of the Netherlands Cancer Institute, the Netherlands. Animals were kept were housed under standard laboratory conditions at the Netherlands Cancer Institute facility and received standard laboratory chow and water *ad libitum*. Wild-type small intestine organoids were derived as previously described (Sato et al., 2009), from Rosa26-Cre^ERT2^::mT/mG (Muzumdar et al., 2007) male and female animals age 9-30 weeks (C57BL/6J background), Lgr5^DTR^ transgenic mice (provided by the Genentech MTA program (Tian et al., 2011)) female animals age 9-19 weeks (C57BL/6J background) and eCadherin-mCFP (Snippert et al., 2010) female animals age 19-22 weeks (mixed background).

#### Isolation intestinal cancer organoids

Small intestine cancer organoids, derived from the small intestine of AKP: Villin-Cre^ERT2^*Apc*^fl/fl^*Kras*^G12D/WT^*Tr53*^fl/R172H^ mice were previously reported (Fumagalli et al., 2017). NOTCH cancer organoids derived from KPN: Villin-Cre^ERT2^*Kras*^G12D/WT^*Trp53*^fl/fl^Rosa26^N1icd/+^ and APN: Villin-Cre^ERT2^*Apc*^fl/+^*Trp53*^fl/fl^Rosa26^N1icd/+^ small intestine primary tumors were previously reported (provided by the ACRCelerate Colorectal Cancer Stratified Medicine Network Consortium (Jackstadt et al., 2019)). All experiments were performed according to UK Home Office regulations (Project License 70/8646), adhered to ARRIVE guidelines and were approved by local animal welfare and the ethical review committee at the University of Glasgow. Mice were housed in conventional cages in an animal room at constant temperature (19–23 °C) and humidity (55% ± 10%) under a 12-h light–dark cycle and were allowed access to standard diet and water *ad libitum*. Male mice (C57BL/6J background) of 7 to 16 weeks of age were induced with a single intraperitoneal injection of 2mg tamoxifen on D0 and either aged until clinical endpoint as evidenced by anemia, hunching and/or weight loss to generate small intestinal tumor organoid lines (APN/KPN), or were sampled on D3 post-induction to generate transformed small intestinal organoids (AKP).

#### Culture of mouse organoids

All lines were cultured in drops of Cultrex PathClear Reduced Growth Factor Basement Membrane Extract Type 2 (Amsbio, 3533-005-02) in murine small intestinal organoids medium containing advanced DMEM/F12 medium (adDMEM/F12; Thermo Fisher Scientific, cat. no. 12634-010), GlutaMAX 1% (Thermo Fisher Scientific, cat. no. 35050-068), HEPES 10mM (Thermo Fisher Scientific, cat. no. 15630-056), 1x Penicillin/streptomycin (10,000 U/ml; Thermo Fisher Scientific, cat. no. 15140-122). B27 2% (Thermo Fisher Scientific, cat. no. 17504-044), N-acetylcysteine 1.25 mM (Sigma-Aldrich, cat. no. A9165), mEGF 50ng/ml (Peprotech, cat. no. 315-09), Noggin and R-spondin1 both 10% (conditioned medium prepared in house).

#### Transduction of organoids

A dual lentiviral vector 3rd generation Tet-regulatory protein expression system was used for doxycycline-inducible expression of MKP5 (custom made by VectorBuilder Inc.). pLentiPGK Hygro DEST H2B-mCerulean3 was a gift from Markus Covert (Addgene plasmid #90234; http://addgene.org/90234; RRID: Addgene_90234) (Kudo et al., 2018). Lentiviral transduction was preformed using standard procedures. In short, lentivirus was produced in HEK293T by co-transfection lentiviral plasmids with helper plasmids pMDLg/pRRE, pRSV-Rev and pMD2.G (gifts from Didier Trono, Addgene plasmids #12251, #12253 and #12259). Viral particles were harvested from cells four days after transfection and concentrated using 50kDa Amicon Ultra-15 Centrifugal Filter Units (Merck, cat#UFC905024). Organoids were dissociated by mechanical disruption and dissolved in 250 μL ENR medium supplemented with 10 μM Y-27632 and Polybrene (8mg/ml) together with concentrated virus. Cells were incubated at 32°C while spinning at 600xG for 1 hour followed by a 4-hour incubation at 37°C before plating in BME2. Selection was carried out from day 3 onward with neomycin, blasticidin or hygromycin.

### Method details

#### Generation of mixed enteroid monolayers

Enteroid monolayers were prepared as described previously (Thorne et al., 2018), in short, single cell suspensions were generated from organoids by mechanical disruption and a digest with TrypLE Express (Thermo Fisher Scientific Cat# 12605-010). Approximately 4000 WT and/or 1000 cancer cells were seeded per well of BME2 coated (0.8mg/ml) 96-well plate in medium supplemented with CHIR-99021 and Y-27632. After 24hrs, cells were washed once and cultured in murine small intestinal organoid medium for the remainder of the experiment. For imaging purposes cells were plated in glass-bottom 96 well SensoPlates (Greiner Bio-One Cat#655892). Small molecule inhibitors were used in the following concentrations: Z-VAD-FMK (50 μM, Bachem Cat# N-1510.0005), CHIR-99021 (3 μM, Tocris Cat#4423), Y-2763 (10 μM, Abmole Cat# M1817).

#### Generation of mixed 3D organoid cultures

3D mixed organoid cultures were prepared as follows; suspensions of small clumps of cells were generated from organoids by mechanical disruption and divided over Eppendorf vials in a 2:1 ratio (WT:cancer). Cells were concentrated by mild centrifugation, the pellet as resuspended in a small volume of murine small intestinal organoids medium and incubated at 37C for 30 minutes. Cell aggregates were plated in BME2 and cultured in murine small intestinal organoids medium. For imaging purposes cells were plated in ibiTreat #1.5 polymer coverslip 96 Well μ-Plate (IBIDI, cat# 89626) or μ-Slide 8 Well chambered slides (IBIDI, cat#80827). Small molecule inhibitors were used in the following concentrations: Z-VAD-FMK (50 μM, Bachem Cat# N-1510.0005), CHIR-99021 (3 μM, Tocris Cat#4423), Valproic acid (1mM, Sigma Cat# PHR1061-1G), Y-2763 (10 μM, Abmole Cat# M1817), JNK-IN-8 (1 μM, Sigma Aldrich, Cat#SML1246), Doxycycline (4.25 μM, Sigma Aldrich, Cat#D9891).

#### Immuno-fluorescence

Enteroid monolayers and 3D organoids were fixed in 4% paraformaldehyde in PBS for 20 minutes followed by a block while permeabilizing in 5% BSA/ 0.2% Triton X-100/ PBS for 30 minutes at room temperature. The stainings were performed overnight at 4°C with the following primary antibodies: anti-Cleaved Caspase-3 (Asp175) (Cell Signaling, #9661), anti-phospho-Histone H3 (Ser10) (Merck-Millipore, #06-570), anti-GFP (Abcam, #ab6673), anti-Phospho-c-Jun (Ser73) (D47G9) (Cell Signaling, #3270), anti-Phospho-JNK1+JNK2 (T183 + Y185) (Abcam, #ab4821) and anti-Ly-6A/E (Sca-1) (Biolegend, cat#108101). Appropriate Alexa Fluor labeled secondary antibodies (ThermoFischer Scientific) were combined with DAPI and/or Phalloidin Alexa Fluor 647 (ThermoFischer Scientific, cat# A22287). For labeling of cells in S-phase, a pulse of 10 μm EdU (5-ethynyl-2′-deoxyuridine) was given one hour prior to fixation and detection was performed according to manufacturer’s guidelines before starting the immunofluorescence staining using Click-iT EdU Cell Proliferation Kit for Imaging Alexa Fluor 647 (ThermoFischer Scientific, cat# C10340).

#### Microscopy

For fixed samples images were collected on an inverted Leica TCS SP8 confocal microscope (Mannheim, Germany) in 12 bit with 25X water immersion objective (HC FLUOTAR L N.A. 0.95 W VISIR 0.17 FWD 2.4 mm).

Sequential imaging of enteriod monolayers was done using the navigator function in LasX software (Leica) on an inverted Leica TCS SP8 confocal microscope (Mannheim, Germany) in 12-bit with 25X water immersion (HC FLUOTAR L N.A. 0.95 W VISIR 0.17 FWD 2.4 mm). Merged images were rotated, aligned and cropped in Photoshop (Adobe).

Time-lapse microscopy of enteroid monolayers was performed on a Leica TCS SP5 confocal microscope (Mannheim, Germany) in 12-bit with 20X dry immersion objective (HCX PL APO CS 20.0x0.70 DRY UV).

Time-lapse microscopy of 3D organoids was done on an inverted Leica TCS SP8 confocal microscope (Figure 1), Leica-based spinning disk confocal microscope with an Andor Dragonfly system, with the Argon-laser of 488nm and the Diode-laser of 561nm using a 40mm pinhole and Andor sCMOS Zyla 4 2p camera (Figure 2), both equipped with a 20X dry immersion objective (HCX PL APO CS 20.0x0.70 DRY UV) or on an AxioObserver widefield microscope (Figures S3B–S3D) (Zeiss) equipped with an Orca FLASH 4.0 V3 grayscale sCMOS-camera (Hamamatsu) and using a 10X dry objective (N.A. 0.30 EC Plan-Neofluar Ph1). Whole drops of organoids were followed up to 90 hours and images were collected in 16-bit with a 6-hour time interval. ZEN software (Zeiss) was used to stitch mosaic images.

#### Flow Cytometry

For gene expression analysis, pure and mixed cells were cultured in 6-well plates and FACS sorted 3 days after mixing on a FACS Jazz system (BD). After a FSC/SSC gate doublets were excluded, live cells (DAPI negative) were sorted based on mTomato (wild-type cells) and Dendra2 (cancer cells) expression. Purity was determined by microscopy.

#### mRNA sequencing

Total RNA was extracted using the standard TRIzol (Invitrogen) protocol and used for library preparation and sequencing. mRNA was processed as described previously, following an adapted version of the single-cell mRNA seq protocol of CEL-Seq (Hashimshony et al., 2012; Simmini et al., 2014). In brief, samples were barcoded with CEL-seq primers during a reverse transcription protocol and pooled after second strand synthesis. The resulting cDNA was amplified with an overnight *In vitro* transcription reaction. From this amplified RNA, sequencing libraries were prepared with Illumina Truseq small RNA primers.

### Quantification and statistical analysis

#### Flow cytometry

Data were manually analyzed using FlowJo 10.6.1 (https://www.flowjo.com/).

#### Microscopy

Imaris software (version 9.3.1, Oxford Instruments) was used for quantification and 3D reconstructions of fixed and time-lapse images. Quantification of cell number, surface and distance was all performed on 3D reconstructed images. In short, individual nuclei and markers (EdU, pH3, pJUN, LGR5) were segmented using the “spots” function and co-localization was determined when relevant (e.g., EdU & pH3). Next, the “surface” function was used to mask individual cell populations and determine the number predefined spots within these populations.

Images and movies were converted to RGB using FIJI, cropped and when necessary corrected for bleed through, smoothened, cropped, rotated and contrasted linearly.

#### Statistical analysis

Statistics were performed using GraphPad Prism. Paired or unpaired t test was used when data showed normal distribution (verified with normality tests, provided by GraphPad Prism), whereas Mann-Whitney U test was used for data that did not display parametric distribution. Adoption of one statistical test or the other is indicated for each experiment in the Figure legend.

#### mRNA sequencing

From paired-end sequencing data read 1 was used to identify the Illumina library index and CEL-Seq sample barcode. After quality control and adaptor removal, read 2 was aligned to the MM10 RefSeq transcriptome using BWA (Li and Durbin, 2010). Reads that mapped equally well to multiple locations were discarded. Reads were quantified with featureCounts to generate read counts for each gene based on the gene annotation from Ensemble. Differential gene expression was analyzed, based on featureCounts results, using DEseq2 version 1.22.2 (Love et al., 2014).

Gene Ontology analysis was performed with Webgestalt (Liao et al., 2019) (http://www.webgestalt.org/), with the parameters; Method: Over-representation Analysis (ORA); Organism: mmusculus; Enrichment Categories: geneontology_Biological_Process; FDR Method: BH; Significance Level: Top 10; Redundancy reduction correction using Weighted set cover algorithm, FDR ≤ 0.05.

The heatmap was generated using Morpheus developed by the Broad Institute (https://software.broadinstitute.org/morpheus) and scaled in Adobe Illustrator.

Gene Set Enrichment Analysis was performed with GSEA software developed by UC San Diego and Broad Institute (Mootha et al., 2003; Subramanian et al., 2005) (https://www.gsea-msigdb.org/gsea/index.jsp). Standard parameters were used with a pre-ranked dataset of differentially expressed genes in wild-type cells (p < 0.05). Gene Sets that were used for comparison were the upregulated genes from the previously published ‘mFetal’ and ‘mRepair’ datasets (Yui et al., 2018).

Transcription Factor target analysis was performed with Webgestalt (Liao et al., 2019) (http://www.webgestalt.org/), with the parameters; Method: Over-representation Analysis (ORA); Organism: mmusculus; Enrichment Categories: network_Transcription_Factor_target; FDR Method: BH; Significance Level: Top 10.

## Supplemental information

Document S1. Figures S1–S7

Document S2. Article plus supplemental information
