# Supplementary material for: Active elimination of intestinal cells drives oncogenic growth in organoids
Source: Cell Rep. 2021 Jul 6;36(1):109307. doi: 10.1016/j.celrep.2021.109307 (PMC8278394; doi:10.1016/j.celrep.2021.109307)
Supplement: Document S1. Figures S1–S7 [file mmc1.pdf]

Cell Reports, Volume *36*

Supplemental information

Active elimination of intestinal cells

drives oncogenic growth in organoids

Ana Krotenberg Garcia, Arianna Fumagalli, Huy Quang Le, Rene Jackstadt, Tamsin Rosemary Margaret Lannagan, Owen James Sansom, Jacco van Rheenen, and Saskia Jacoba Elisabeth Suijkerbuijk

Krotenberg Garcia et al, Figure S1

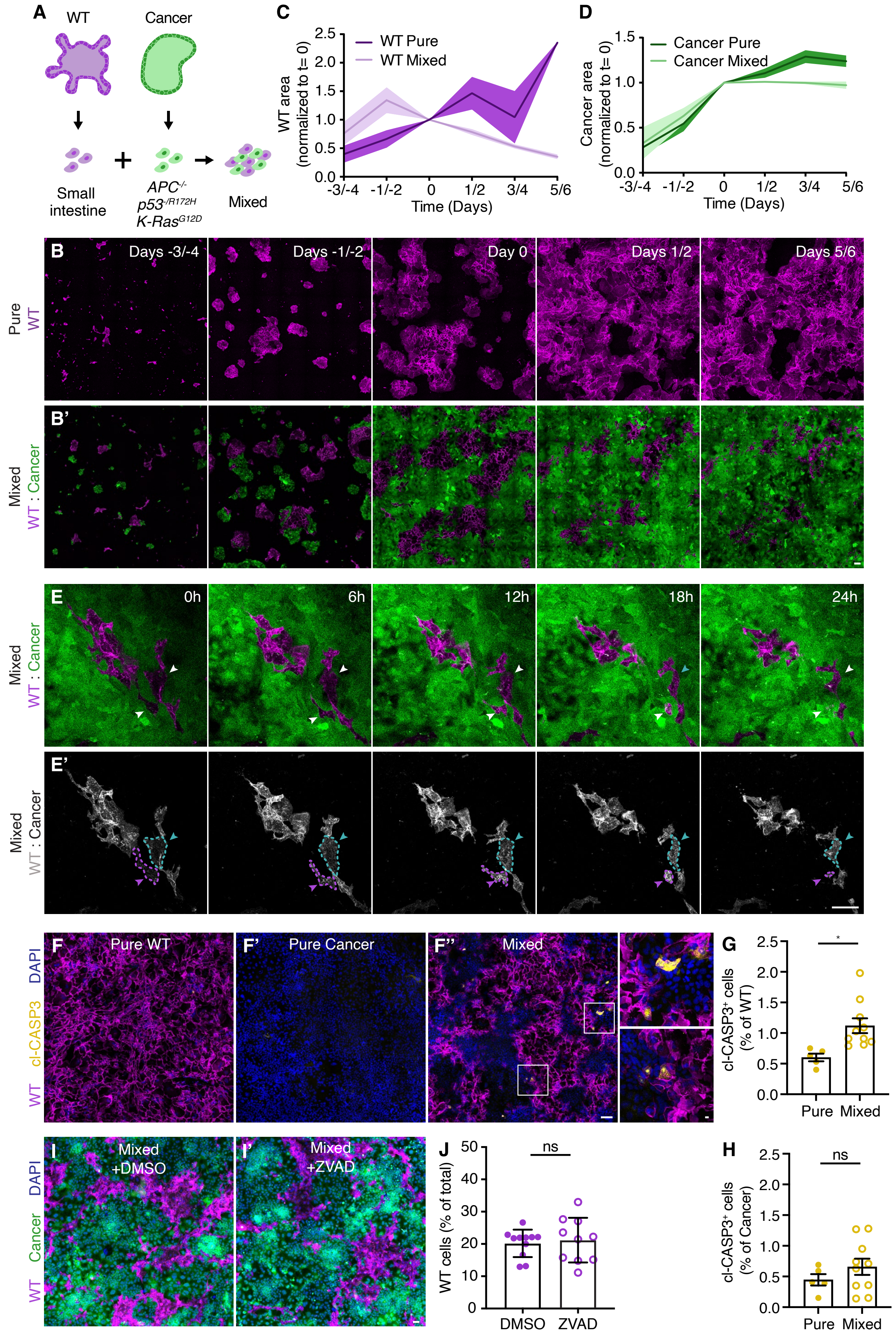

(legends on next page)

Figure S1 - Wild-type small intestine cells are eliminated by cancer cells in enteroid monolayers. Related to Figures 1 and 2.

A) Schematic representation of a model for cell competition in murine enteroid monolayers.

B-D) Representative pictures of sequential imaging of enteroid monolayers in pure (B) and mixed (B') conditions and quantification of the surface covered by wild-type (C) or cancer (D) populations over time normalized to Day 0 (Mean ±SEM). Day 0 is the moment a full monolayer is formed in mixed conditions.

E) Representative images of time-lapse series of a competing enteroid monolayer, arrow heads in (E') indicate examples of wild-type cells that are shrinking (cyan) and being eliminated (magenta).

F-H) Representative confocal images of pure wild-type (F) pure cancer (F') and mixed (F'') enteroid monolayers. Apoptotic cells are marked by cl-CASP3 (yellow). The insets display a 5.75x magnification of the area in the white box. G-H) Quantification of the cl-CASP3+ cells relative to the total wild-type (G) and cancer (H) cell population, each dot represents one imaged well (Mean ±SEM, unpaired t-test, two-tailed, p=0.0128 (G), p=0.3092, n=5 & 10 wells).

I-J) Representative confocal images of control (I) and Z-VAD-FMK (I') treated enteroid monolayers. J) Quantification of the number of wild-type cells relative to the cell population, each dot represents one imaged well (Mean ±SEM, unpaired t-test, two-tailed, p=0.6926, n=11 & 10 wells).

Scale bars = 100µm, excluding magnifications in (F) where scale bar = 10µm.

Krotenberg Garcia et al, Figure S2

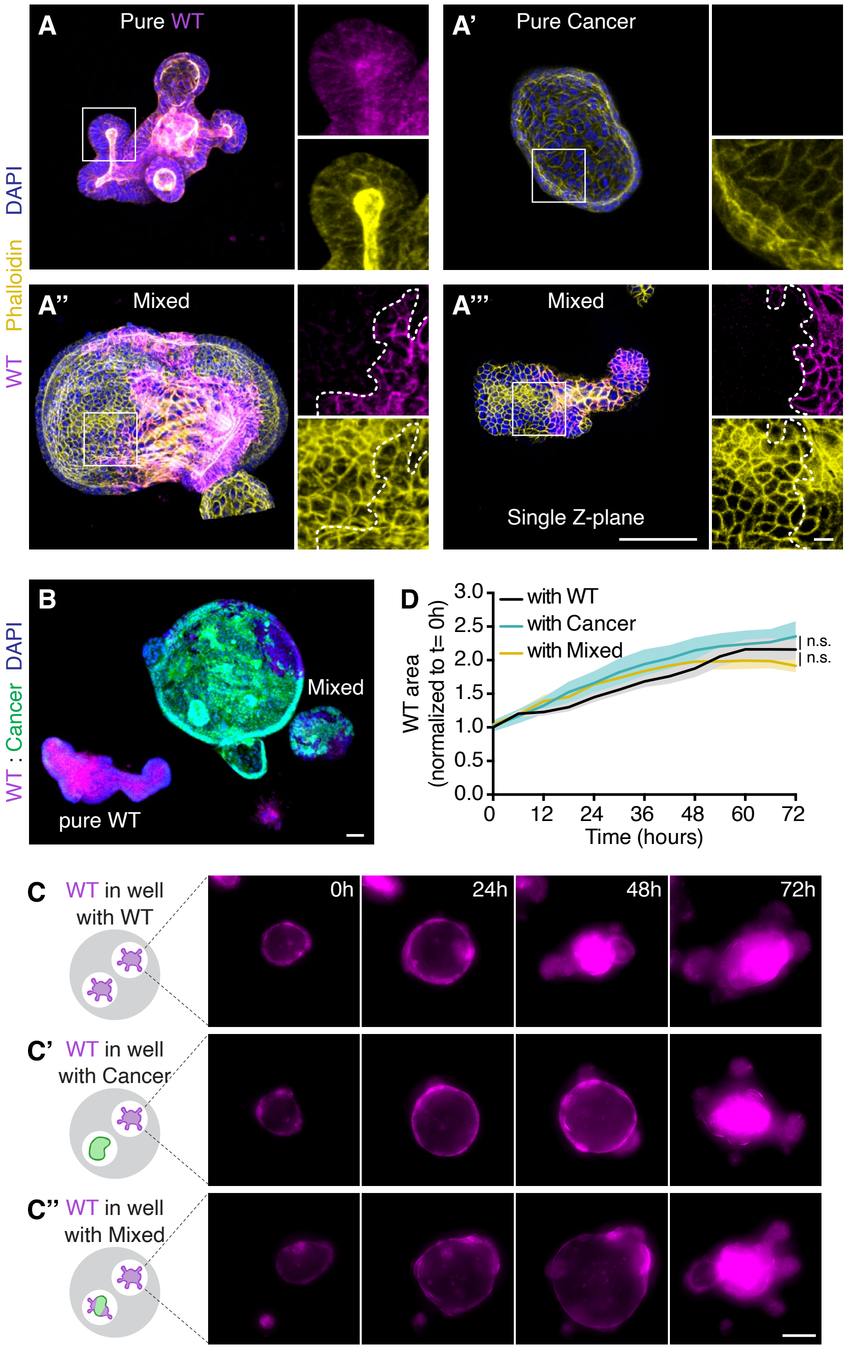

Figure S2 - Short-range communication is essential for cell competition. Related to Figure 2.
A) Representative 3D-reconstructed confocal images of pure WT (A), pure cancer (A') and mixed (A'') organoids, and a single Z-plane of A'' (A'''). The actin cytoskeleton is stained with Phalloidin (yellow), nuclei with DAPI (blue) and borders between wild-type and cancer cells are indicated by dashed lines. The insets display a 2.5x magnification of the area in the white box.
B) Representative confocal image of a mixed culture containing a pure WT organoid, nuclei are visualized with DAPI (blue).
C-D) Representative images from live-imaging of pure WT organoids co-cultured with pure WT (C), pure cancer (C') or mixed (C'') organoids and quantification of the area covered by wild-type cells within indicated organoids (D) normalized to the start of the time-lapse (Mean ±SEM, 2-way ANOVA, multiple comparisons, n=18 organoids for each condition, 'WT in WT' vs. 'WT in cancer' p= 0.5453, 'WT in WT' vs. 'WT in Mix' p= 0.9689).
Scale bars = 100µm, excluding magnifications in (A) where scale bar = 10µm.

Krotenberg Garcia et al, Figure S3

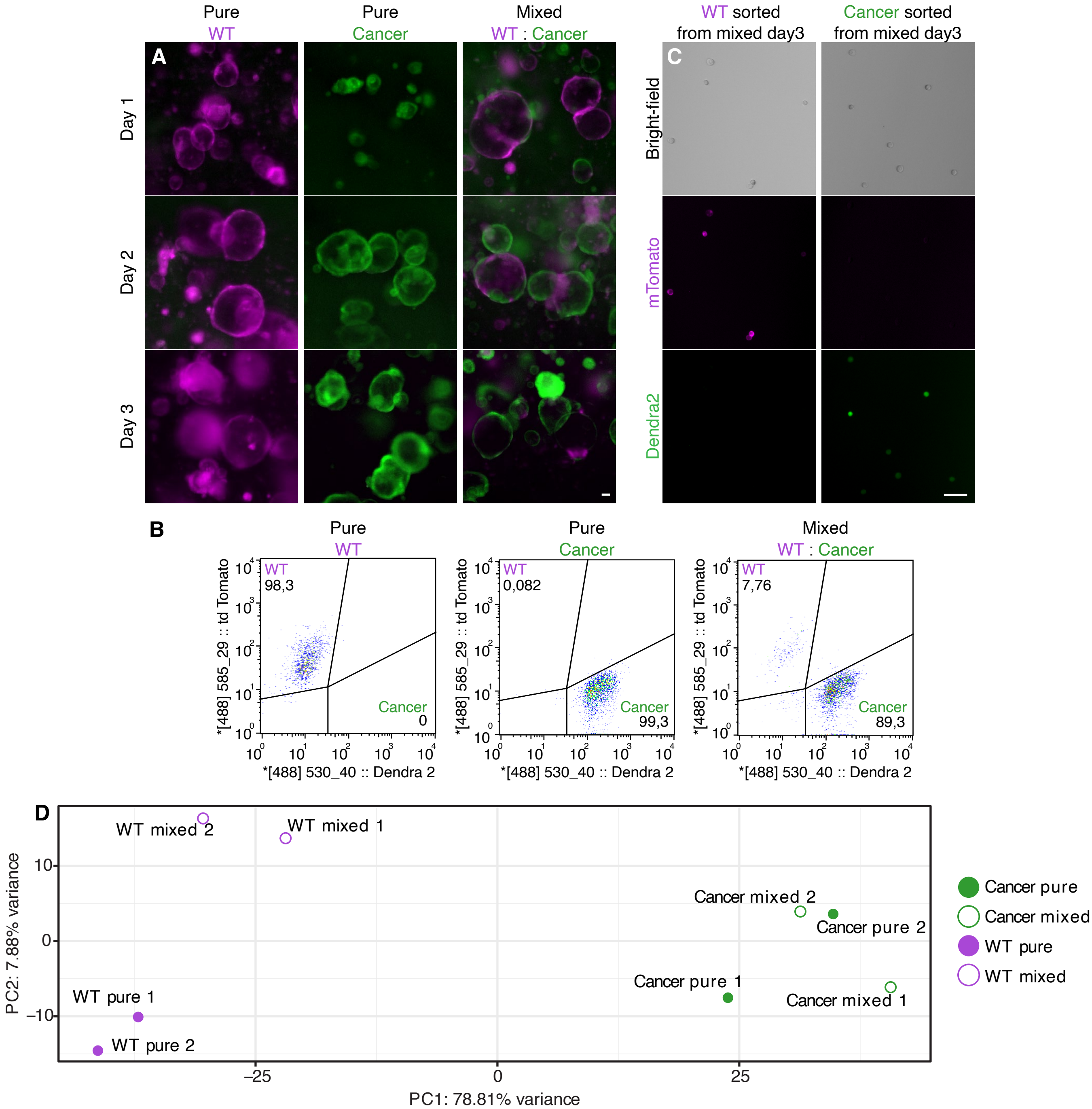

E

GeneSet	mFetal
Enrichment Score (ES)	0.4131525
Normalized Enrichment Score (NES)	2.0948782
Nominal p-value	0.000
FDR q-value	0.000
FWER p-Value	0.000

F

GeneSet	mRepair
Enrichment Score (ES)	0.43847787
Normalized Enrichment Score (NES)	1.8192958
Nominal p-value	0.0011933175
FDR q-value	5.030181E-4
FWER p-Value	0.001

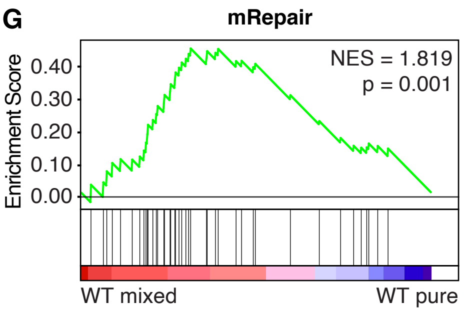

(legends on next page)

Figure S3 - Cell competition induces a fetal-like state in WT cells. Related to Figure 4.

A-C) Flow cytometry sorting of wild-type and cancer cells from pure and mixed cultures. A) Representative images of pure and mixed cultures 1, 2 and 3 days after plating. B-C) Analysis of cells after sorting, graphs in B show an analysis of 10.000 cells, numbers in the corners display the percentage of sorted cells. Representative images of sorted cells are shown in (C) Scale bars = 50µm. D-G) Gene expression analysis of wild-type and cancer cells in pure and mixed conditions. D) displays a principal component analysis of all sample. D) Parameters of a gene Set Enrichment Analysis showing enrichment of a fetal signature (Yui et al., 2018) in mixed wild-type cells. F-G) Parameters and graph of a gene Set Enrichment Analysis showing enrichment of a repair signature (Yui et al., 2018) in mixed wild-type cells.

Krotenberg Garcia et al, Figure S4

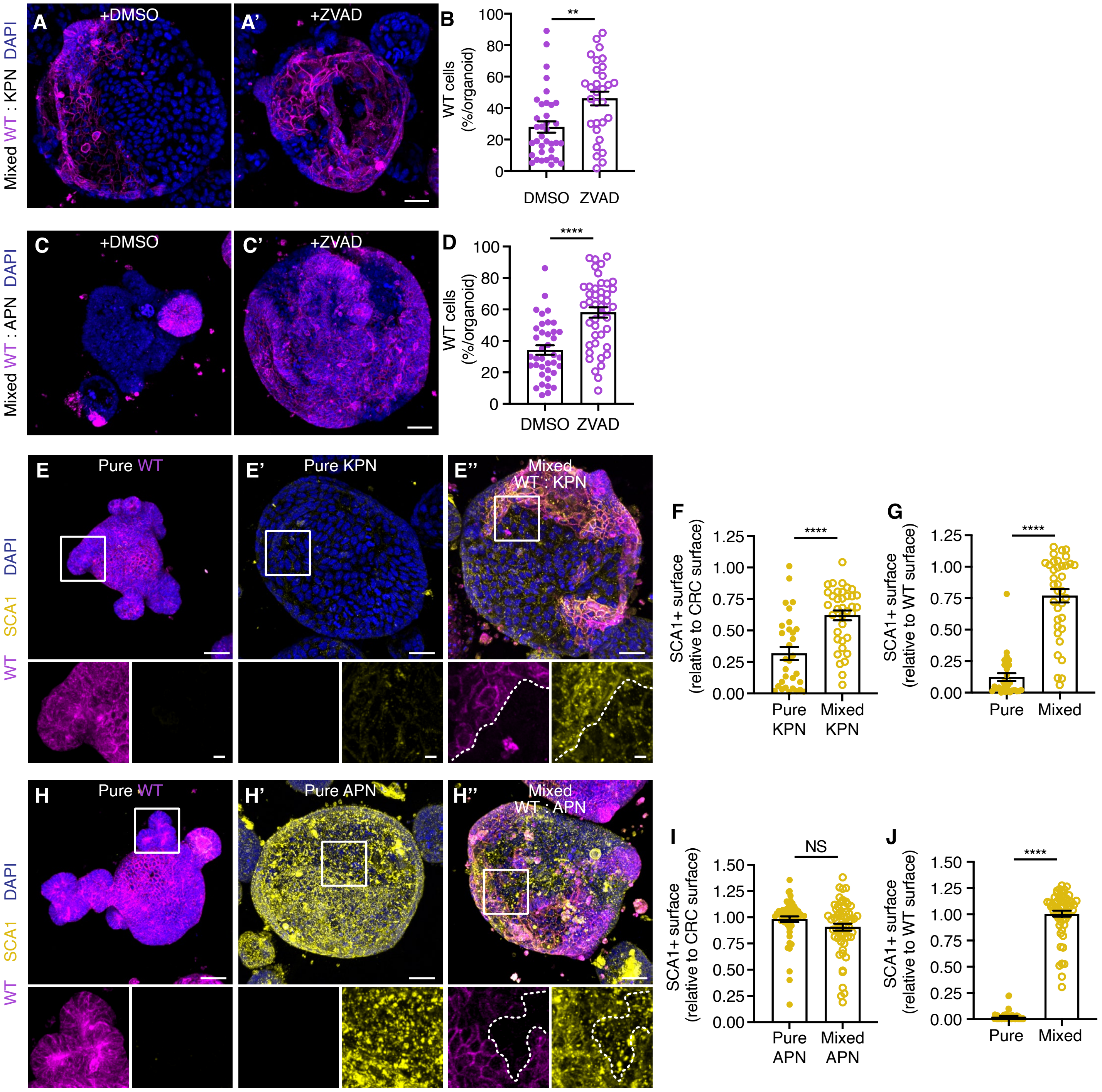

(legends on next page)

Figure S4 - Multiple types of intestinal cancer compete with WT cells. Related to Figures 2 and 4.

A-B) Representative 3D-reconstructed confocal image of control (A) and apoptosis inhibited (A') mixed organoids formed by KPN cancer and wild-type intestinal cells, nuclei are stained with DAPI (blue), and quantification of the percentage of wild-type cells contributing to mixed organoids (B), each dot represents one organoid (Mean ±SEM, unpaired t-test, two-tailed, p=0.019, n=36 & 30 organoids).

C-D) Representative 3D-reconstructed confocal image of control (C) and apoptosis inhibited (C') mixed organoids formed by APN cancer and wild-type intestinal cells, nuclei are stained with DAPI (blue), and quantification of the percentage of wild-type cells contributing to mixed organoids (D), each dot represents one organoid (Mean ±SEM, unpaired t-test, two-tailed, p<0.0001, n=38 & 44 organoids).

E-G) Representative 3D-reconstructed confocal images of pure WT (E), pure KPN cancer (E'), mixed KPN (E'') organoids and quantification of the SCA1+ surface relative to the total KPN cancer (F) or wild-type (G) surface area. The organoids were stained for SCA1 (yellow), nuclei are visualized with DAPI (blue). The insets display a 2.5x magnification of the area in the white box. Each dot in (F) and (G) represent one organoid (Mean ±SEM, Non-parametric, ANOVA, multiple comparisons: p<0.0001, n=31 & 36 organoids (F); p<0.0001, n=28 & 36 organoids (G)).

H-J) Representative 3D-reconstructed confocal images of pure WT (H), pure APN cancer (H'), mixed APN (H'') organoids and quantification of the SCA1+ surface relative to the total APN cancer (I) or wild-type (J) surface area. The organoids were stained for SCA1 (yellow), nuclei are visualized with DAPI (blue). The insets display a 2.5x magnification of the area in the white box. Each dot in (I) and (J) represent one organoid (Mean ±SEM, Non-parametric, ANOVA, multiple comparisons: p=0.0939, n=54 & 58 organoids (I); p<0.0001, n=50 & 58 organoids (J)).

Scale bars = 50µm, excluding magnifications in (E and H) where scale bar = 10µm

Krotenberg Garcia et al, Figure S5

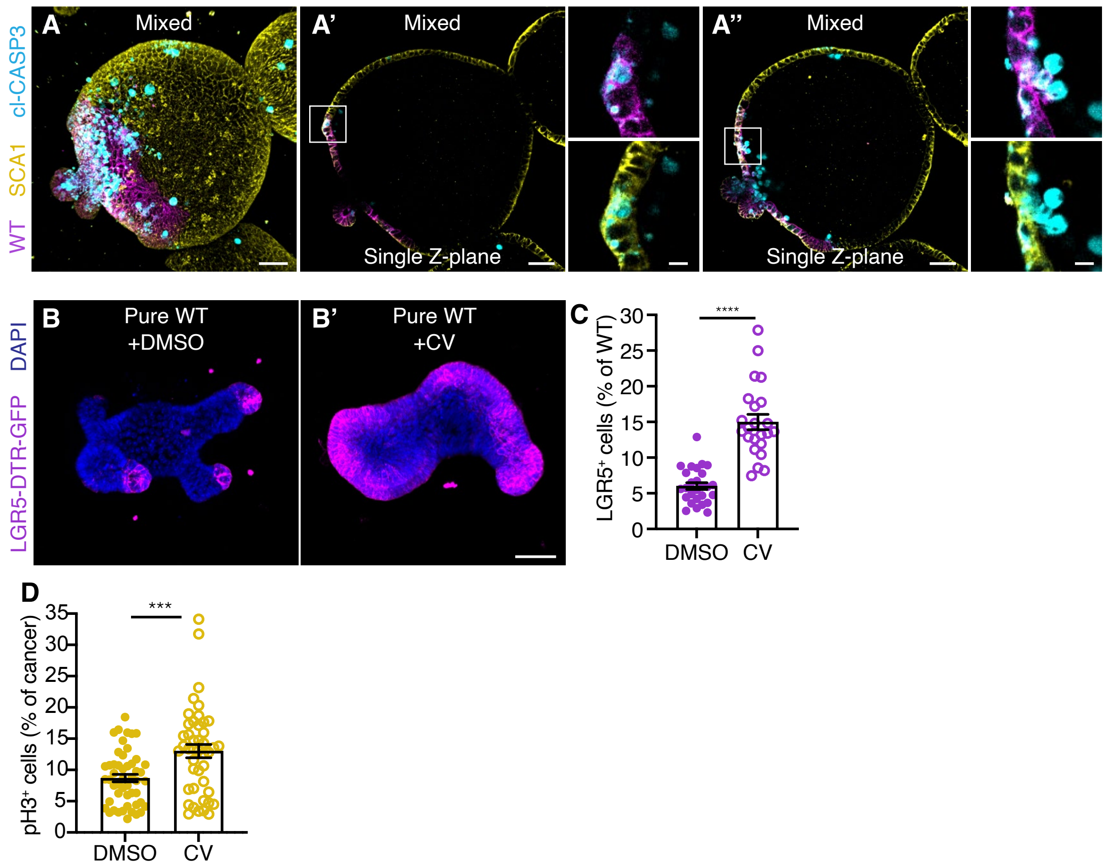

Figure S5 - Increased stemness prevents cell competition. Related to Figures 2 and 5.
A) Representative 3D-reconstructed (A) and single Z-plane (A' and A'') confocal images of a mixed organoid. The organoids were stained for cl-CASP3 (cyan) and SCA1 (yellow). The insets display a 3.5x magnification of the area in the white box. Organoid is from the same dataset used in panel 2E.
B) Representative 3D-reconstructed confocal images of control (B) and CV treated (B') pure WT organoids. LGR5+ Intestinal stem cells (magenta) and nuclei (blue) are visualized.
(C) Graph displays the number of LGR5+ cells relative to total number of wild-type cells, each dot represents one organoid (Mean ±SEM, unpaired T-test, two-tailed, $p<0.0001$, n=27 & 23 organoids). Displayed DMSO control organoids are from the same dataset used in panel 5B.
D) Graph displays the number of pH3+ cells in pure organoids relative to the total number of cancer cells, each dot represents one organoid (Mean ±SEM, one-way ANOVA, multiple comparisons, $p=0.0005$, n=50 & 44 organoids).
Scale bars = 50µm

Krotenberg Garcia et al, Figure S6

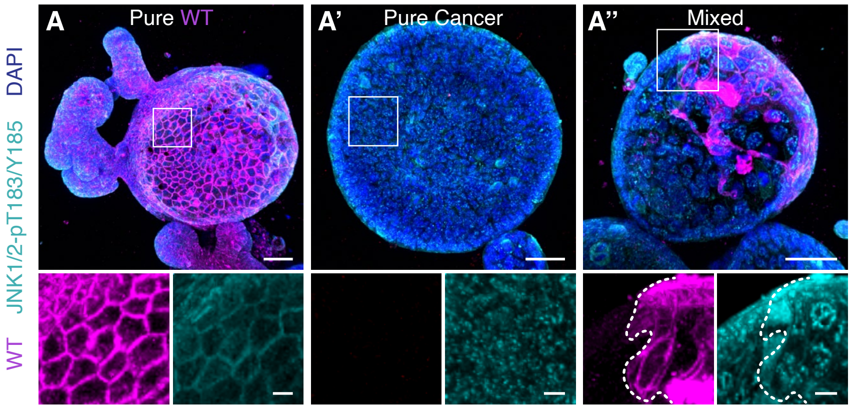

Figure S6 - JNK signaling drives cell competition. Related to Figure 6.
Representative 3D-reconstructed confocal images of pure WT (A), pure cancer (A'), mixed (A'') organoids, stained for activated JNK1/2-pT183/Y185 (cyan), nuclei are visualized with DAPI (blue). The insets display a 2.5x-3.5x magnification of the area in the white box.

Krotenberg Garcia et al, Figure S7

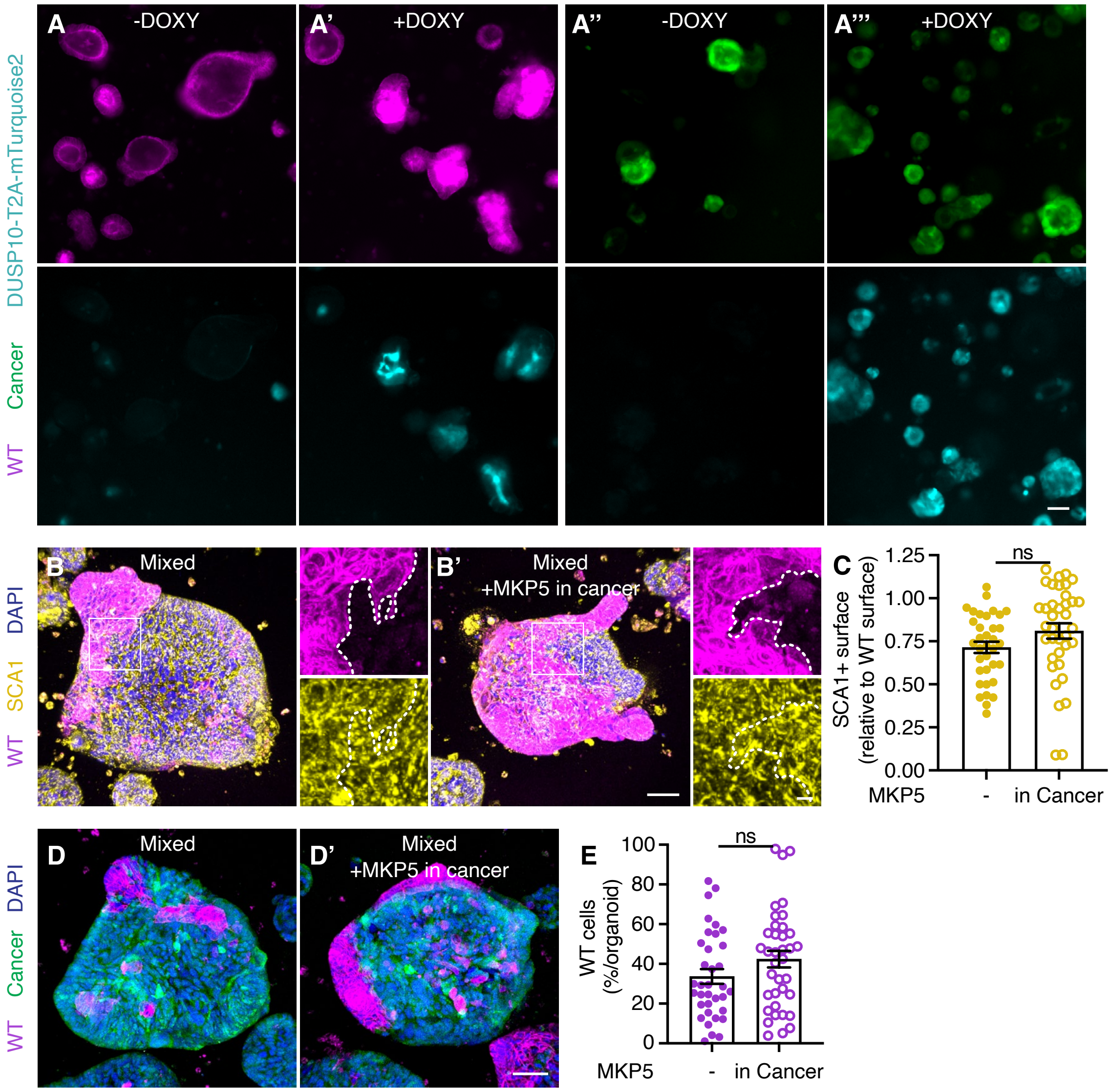

Figure S7 - JNK activity in wild-type cells is required for cell competition. Related to Figure 7.
A) Representative images of control (A) and doxycycline treated (A') organoids formed by TET-inducible MKP5 wild-type cells and control (A'') and doxycycline treated (A''') organoids formed by TET-inducible MKP5 cancer cells, co-expression of mTurquoise2 (Cyan) is shown.
B-C) Representative 3D-reconstructed confocal images of control (B) and doxycycline treated (B') mixed organoids formed by TET-inducible MKP5 cancer and control wild-type cells. The organoids were stained for SCA1 (yellow), nuclei are visualized with DAPI (blue). The insets display a 2.5x magnification of the area in the white box. C) Quantification of the SCA1+ surface relative to the total wild-type surface area, each dot represents one organoid (Mean ±SEM, ANOVA, multiple comparisons, p=0.2337, n=35 & 37 organoids).
D-E) Representative 3D-reconstructed confocal image of control (D) and doxycycline treated (E') mixed organoids formed by TET-inducible MKP5 cancer and control wild-type cells, nuclei are stained with DAPI (blue), and quantification of the percentage of wild-type cells contributing to mixed organoids (E), each dot represents one organoid (Mean ±SEM, ANOVA, multiple comparisons, p=0.2892, n=35 & 37 organoids).
Scale bars = 50µm, excluding magnifications in (B) where scale bar = 10µm
